# Association between pregnancy at enrollment into HIV care and loss to care among women in the Democratic Republic of Congo, 2006-2013

**DOI:** 10.1371/journal.pone.0195231

**Published:** 2018-04-02

**Authors:** Jonathan Ross, Andrew Edmonds, Donald R. Hoover, Qiuhu Shi, Kathryn Anastos, Patricia Lelo, Frieda Behets, Marcel Yotebieng

**Affiliations:** 1 Department of Medicine, Division of General Internal Medicine, Department of Medicine, Albert Einstein College of Medicine / Montefiore Medical Center, Bronx, NY, United States of America; 2 Department of Epidemiology, Gillings School of Global Public Health, The University of North Carolina at Chapel Hill, Chapel Hill, NC, United States of America; 3 Department of Statistics and Biostatistics, Rutgers University, New Brunswick, NJ, United States of America; 4 Department of Epidemiology and Community Health, New York Medical College, Valhalla, NY, United States of America; 5 Kalembelembe Pediatric Hospital, Kinshasa, The Democratic Republic of Congo; 6 College of Public Health, Division of Epidemiology, The Ohio State University, Columbus, OH, United States of America; Cameroon Baptist Convention Health Services, CAMEROON

## Abstract

**Background:**

Loss to care is high among asymptomatic HIV-infected women initiated on antiretroviral therapy (ART) during pregnancy or in the postpartum period. However, whether pregnancy itself plays a role in the high loss to care rate is uncertain. We compared loss to care over seven years between pregnant and non-pregnant women at enrollment into HIV care in the Democratic Republic of Congo (DRC).

**Methods:**

We conducted a retrospective analysis of all ART-naive women aged 15–45 initiating HIV care at two large clinics in Kinshasa, DRC, from 2007–2013. Pregnancy status was recorded at care enrollment. Patients were classified as having no follow-up if they did not return to care after the initial enrollment visit. Among those with at least one follow-up visit after enrollment, we classified patients as lost to care if more than 365 days had passed since their last clinic visit. We used logistic regression to model the association between pregnancy status and no follow-up, and Cox proportional hazards regression to model the association between pregnancy status and time to loss to care.

**Results:**

Of 2175 women included in the analysis, 1497 (68.8%) were pregnant at enrollment. Compared to non-pregnant women, pregnant women were less likely to be over 35 years of age (19.1% vs. 31.9%, *p*<0.0001) and less likely to be in WHO stage III or IV (9.0% vs. 26.3%, *p*<0.0001). Among pregnant women, 106 (7.1%) were not seen after enrollment, versus 25 (3.7%) non-pregnant women (adjusted odds ratio 2.01, 95% CI 1.24–3.24). Of the 2,044 women with at least one follow-up visit, 46.5% of pregnant women and 46.7% of non-pregnant women were lost to care by 5 years; hazards of loss to care were similar for pregnant and non-pregnant women (adjusted hazard ratio 1.08, 95% CI 0.93–1.26).

**Conclusions:**

In this large cohort of HIV-infected women, patients pregnant at care enrollment were more likely to never return for follow-up. Among those who attended at least one follow-up visit, loss to care was not different between pregnant and non-pregnant women, suggesting that pregnancy itself may not be the main driver of the high attrition observed in this cohort.

## Introduction

Of 1.4 million pregnant women living with HIV globally, over 90% reside in sub-Saharan Africa [1, 2]. Without any intervention, 20−45% of pregnant women will transmit HIV to their infants during pregnancy, labor/delivery, or breastfeeding [3]. Antiretroviral therapy (ART) use during pregnancy and breastfeeding dramatically reduces mother-to-child HIV transmission and improves women and infant outcomes [4, 5]. The past decade has seen enormous scale up of prevention of mother-to-child HIV transmission (PMTCT) services in sub-Saharan Africa, including ART access [6]. However, suboptimal retention of pregnant women along the PMTCT cascade exists, with up to 48% of women not retained through 6 months post-delivery [7–9]. Under World Health Organization (WHO) guidelines recommending lifelong combination ART for all pregnant and breastfeeding HIV infected women, referred to as Option B+ [10], about a sixth of pregnant women initiating ART do not return for a follow-up visit and overall loss to care of women initiating HIV care when pregnant remains high [11–13].

Uncertainty exists about factors driving high loss to care among pregnant women initiating HIV care. Compared to non-pregnant women, pregnant women initiating HIV care are younger and have less advanced HIV disease [14], factors that have been associated with poor retention [15, 16]. After adjusting for these characteristics, multiple large investigations still reported greater loss to care among pregnant women [11–13, 17]. However, interpreting these findings is difficult as different ART eligibility criteria exist for pregnant and non-pregnant women in these studies. Few studies have compared retention in care of pregnant and non-pregnant women before implementation of the 2013 WHO guidelines; those investigations only included patients initiating ART. It is therefore unclear whether the high loss to care observed among pregnant versus non-pregnant women in these studies resulted from lack of patient readiness to start ART, or factors secondary to pregnancy itself.

Most sub-Saharan Africa nations have already adopted Option B+, and nearly all are beginning to provide ART to all people living with HIV regardless of CD4 count. Understanding whether pregnancy at care enrollment is a main predictor of the loss to care observed following the implementation of Option B+ is essential to developing and targeting interventions aimed at maintaining the broader population of people living with HIV in care. This study’s goal was thus to compare loss to HIV care by pregnancy status at enrollment at two sites in the Democratic Republic of Congo (DRC) from 2007–2013, a period prior to implementation of lifelong ART for pregnant and breastfeeding women living with HIV. We hypothesized that women who were pregnant at enrollment would have increased loss to care compared to women who were not pregnant.

## Methods

We performed a retrospective cohort study of women living with HIV enrolling in HIV care from 2007–2013 at two sites in Kinshasa, DRC that are affiliated with the Central Africa International Epidemiologic Databases to Evaluate AIDS (CA-IeDEA) Consortium. CA-IeDEA is a multi-country project that collects secondary data from patients receiving HIV care and treatment in the Central African region. Patient data from sites participating in CA-IeDEA are de-identified at the clinic level prior to entry in the cohort database. All research was conducted according to the principles of the Declaration of Helsinki and approved by the Kinshasa School of Public Health Ethics Committee and the Institutional Review Board of the Albert Einstein College of Medicine, both of which waived written or verbal patient consent because the data were de-identified prior to analysis.

### Setting

Data were collected at two comprehensive HIV care programs serving patients at Kalembelembe Pediatric Hospital (KLL) and Bomoi Healthcare Center (BHC) in Kinshasa, DRC [18, 19]. The DRC is a large, Central African country with a population of over 80 million and an overall HIV prevalence of 0.8% [20]. An estimated 200,000 Congolese women aged 15–49 are HIV-infected. Up to 15% of pregnancies among HIV-infected women result in HIV transmission to infants [20]. From 2013 Demographic and Health Surveys in DRC [21], roughly 9 in 10 mothers in DRC and 96% in Kinshasa attended at least one antenatal care visit before their most recent delivery. Breastfeeding is virtually universal in DRC; 9 out of 10 infants still breastfeed at 1 year [22, 23]. From its origins in the early 2000s until 2010, the DRC PMTCT program was based on provision of a single intra-partum dose of nevirapine, with the addition of daily AZT/3TC until 7 days postpartum recommended starting in 2006 [24, 25]. Option A–the provision of zidovudine starting as early as 14 weeks through delivery for women ineligible for ART [26]–was introduced in 2009 and fully implemented in DRC by 2010; this remained the standard of care until the implementation of Option B+ in 2015.

### Population and clinical procedure

We included all ART-naïve women aged 15–45 years who enrolled in HIV care from January 1, 2007 through July 31, 2013. Women were diagnosed with HIV at the two sites or referred to these sites from affiliated PMTCT sites. Patients enrolled into HIV care at their first post-diagnosis visit at KLL or BHC. At this visit, data were collected on pregnancy status, weight, age, WHO clinical stage, and CD4 count. Decisions to start ART were based on WHO clinical stage (III or IV), CD4 count (≤200 cells/mm^3^ prior to 2010 or ≤350 cells/mm^3^ after 2010) or provider discretion. Pregnant women ineligible for ART received either a single intrapartum dose of nevirapine with or without daily AZT/3TC until 7 days postpartum (from 2007–2009), or a regimen of zidovudine during pregnancy (2010–2013) and were considered not to have used ART. Scheduled follow-up differed by calendar period and whether or not patients had initiated ART. From 2007–2009, patients on ART were typically scheduled for monthly visits, and patients not on ART were typically seen quarterly. In subsequent years, patients on ART were initially seen monthly, but once stable on ART were scheduled quarterly; those not on ART were seen biannually. Though tracking practices were not uniform throughout the study period, typically, for patients who did not attend scheduled visits, up to three phone contacts were attempted, followed by a home visit.

### Outcomes and predictor variables

Two primary outcomes were considered in this analysis: no follow-up and loss to care. Patients were classified as having no follow-up if they did not return to care after the initial enrollment visit, and were not included in analyses of loss to care. We defined loss to care as an interval of more than 365 days since last clinic visit to avoid misclassification among patients scheduled for infrequent (biannual) visits. In a sensitivity analysis, we also examined loss to care using a definition of 182 days since last clinic visit. Other covariates included: ART initiation within three months of enrollment (yes or no); pregnancy status; baseline age (categorized as 15–24, 25–34, or 35–45 years at enrollment); baseline CD4 count (classified as <200, 201–350, or >350 cells/mm^3^); and WHO clinical stage (I or II, III or IV, missing) each of these obtained at any visit up to 30 days after enrollment.

### Statistical analyses

Baseline characteristics of pregnant and non-pregnant women were compared using chi-square tests for categorical and Mann-Whitney tests for continuous variables. For the outcome of no follow-up, we used bivariate and multivariable logistic regression models respectively to generate crude and adjusted odds ratios (ORs) with 95% confidence intervals (CIs) comparing proportions of women with no follow-up after enrollment by pregnancy status at enrollment. For those who returned for at least one post-enrollment visit, Kaplan-Meier survival analyses were used to compare proportions of pregnant and non-pregnant women lost to care over follow-up time. We then used Cox proportional hazard models to generate hazard ratios (HRs) with 95% CIs comparing loss to care by pregnancy status at enrollment. For these analyses, patients were censored at the date of their last clinic visit (if they had one of the following events: lost to care, died, or transferred clinics) or 365 days before the database closing date of August 1, 2013 (if they did not have an event). We also conducted a sensitivity analysis exploring whether the association between pregnancy status at enrollment and loss to care differed when limiting follow-up time to the first 2 years after enrollment, when pregnancy might be expected to have the strongest effect. For this analysis, we utilized a 182-day loss to care definition and censored patients who did not have an event by 24 months after their enrollment date. Data were analyzed using SAS 9.3 (SAS Institute Inc., Cary, NC). Statistical significance was two-sided at *p***<**0.05.

## Results

Of the 2175 women enrolled in care at the two clinics from 2007 through 2013, 982 (45.1%) enrolled from 2007–2009 and 1173 (54.9%) from 2010–2013. At enrollment, 1497 (68.8%) were pregnant, median age was 30 years (interquartile range [IQR] 26–34), and 312 (14.3%) were in WHO clinical stage III or IV (**Table 1**). Median CD4 count at enrollment was 356 cells/mm^3^ (IQR 215–534). A total of 131 patients (6.0%) did not return after the enrollment visit.

**Table 1:** Baseline demographic and clinical characteristics of HIV-infected women who received care at Kalembelembe Pediatric Hospital and Bomoi Healthcare Center in Kinshasa, DRC, 2007–2013 (N = 2175).

Characteristic	Total (n = 2175)	Pregnant (n = 1497)	Not pregnant (n = 678)	*P*
Year of enrollment, n (%)				<0.0001^^^
2007–2009	982 (45.1)	768 (51.3)	214 (31.6)	
2010–2013	1193 (54.9)	729 (49.7)	464 (68.4)	
Age, n (%)				<0.0001^^^
15–24	372 (17.1)	273 (18.2)	99 (14.6)	
25–34	1301 (59.8)	938 (62.7)	363 (53.5)	
35–45	502 (23.1)	286 (19.1)	216 (31.9)	
WHO clinical stage, n (%)				<0.0001^^^
Stage I or II	1848 (85.0)	1351 (90.2)	497 (73.3)	
Stage III or IV	312 (14.3)	134 (9.0)	178 (26.3)	
Missing	15 (0.7)	12 (0.8)	3 (0.4)	
CD4 count, median cells/mm^3^ (IQR)	356 (215–534)	349 (224–515)	366 (196–574)	0.31^&^
ART initiated within 3 months of enrollment, n (%)	802 (36.9)	506 (33.8)	296 (43.7)	<0.0001^^^
Median years of follow-up (IQR)	4 (3–6)	4 (3–5)	5 (3–6)	<0.0001^&^
No follow-up after enrollment, n (%)	131 (6.0)	106 (7.1)	25 (3.7)	<0.0001^^^
Lost to care, n (%)*	860 (42.1)	572 (41.0)	288 (43.8)	0.60^^^
Transferred to another facility	32 (1.5)	21 (1.4)	11 (1.6)	0.69^^^
Died	91 (4.2)	56 (3.7)	35 (5.2)	0.13^^^

DRC = Democratic Republic of Congo.

IQR = interquartile range.

WHO = World Health Organization.

ART = antiretroviral therapy.

^ represents probability of observed result using chi-squared test of independence.

^&^ represents probability of observed result using Wilcoxon Rank-Sum test.

* denominators do not include the 131 women (106 pregnant and 25 not pregnant) who did not return after the enrollment visit.

Compared to non-pregnant women, pregnant women at enrollment were less likely to: be over 35 years of age (19.1% vs. 31.9%, *p*<0.0001), enroll in care during 2010–2013 compared to 2007–2009 (49.7% vs. 68.4%, *p*<0.0001), and be in WHO clinical stage III or IV (9.0% vs. 26.3%, *p*<0.0001). Notably, baseline CD4 counts did not statistically differ between the two groups (p = 0.31).

**Fig 1:**
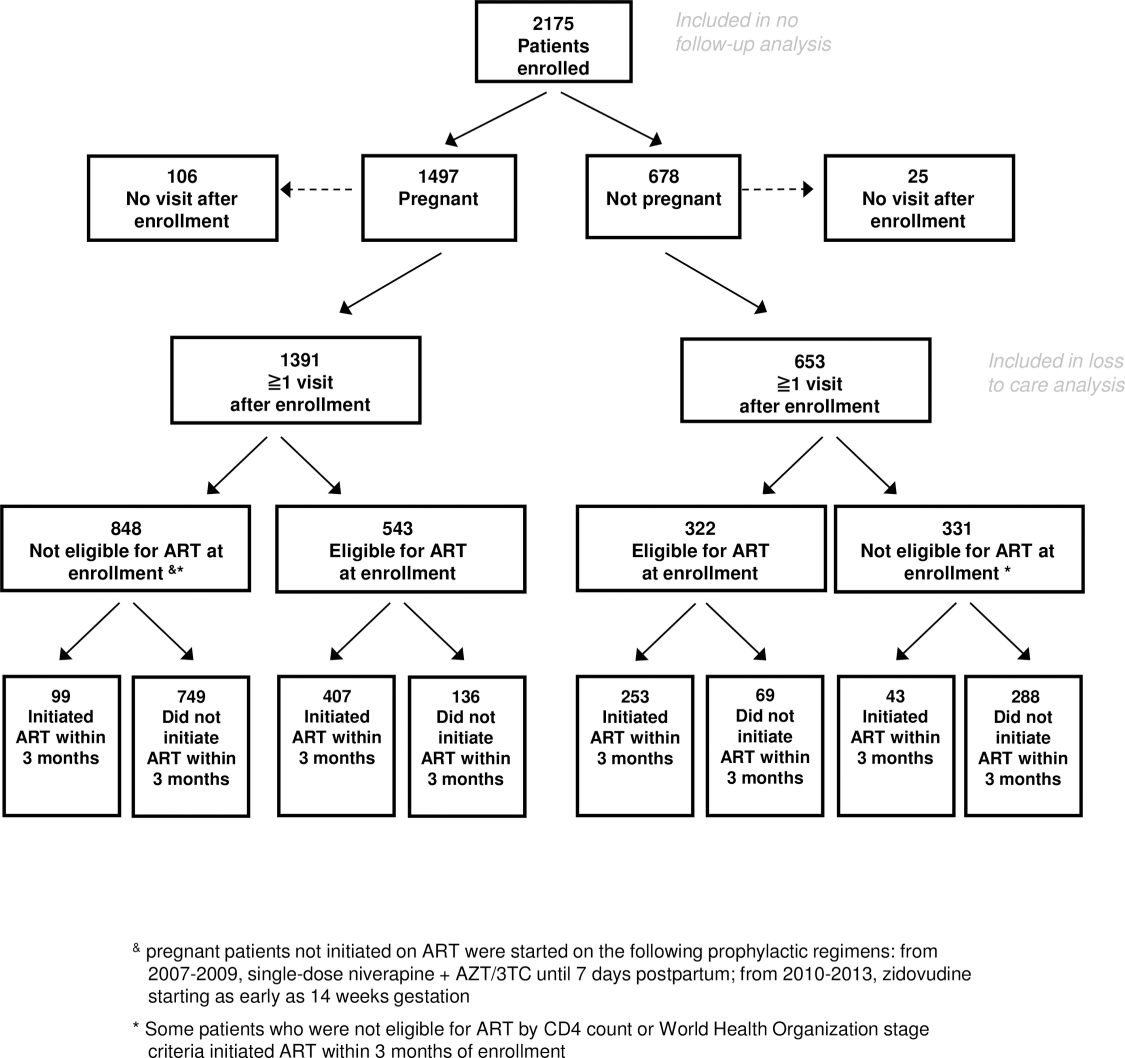
Flow chart describing study population.

Among 2,044 patients who returned for at least one visit after enrollment, 865 (42.3%) were eligible for ART at enrollment based on CD4 count and/or WHO stage, of whom 660 (76.3%) started ART within 3 months (**Fig 1**). An additional 142 patients initiated ART within 3 months of enrollment, and another 465 patients initiated ART beyond 3 months. In total, 1267 patients (58.3%) initiated ART during the follow-up period. Among patients meeting CD4 and/or WHO stage criteria for ART initiation, similar proportions of pregnant and non-pregnant women initiated ART within three months after enrollment into care (75.0% vs 78.6%, *p* = 0.23).

### No follow-up

Of 1497 pregnant women enrolled in HIV care, 106 (7.1%) were not seen after enrollment, compared to 25 (3.7%) of 678 non-pregnant women (OR comparing pregnant to non-pregnant women 1.99, 95% CI 1.27–3.11), *p*<0.01). In logistic regression analysis adjusted for year of enrollment, age at enrollment, baseline CD4 count, and WHO clinical stage, pregnant women were about twice as likely not to be seen after enrollment (adjusted OR 2.11, 95% CI 1.24–3.24) (**Table 2**).

**Table 2:** Associations of demographic and clinical characteristics at enrollment and having no follow-up* after enrollment (N = 2175).

Characteristic	No follow-up* (n = 131)	Followed-up* (n = 2044)	Crude OR (95% CI)	Adjusted OR (95% CI)
Pregnant				
No	25 (3.7)	653 (96.3)	Ref.	Ref.
Yes	106 (7.1)	1391 (92.9)	1.99 (1.27, 3.11)	2.01 (1.24, 3.24)
Age (years)				
15–24	36 (9.7)	336 (90.3)	Ref.	Ref.
25–34	76 (5.8)	1225 (94.2)	0.58 (0.38, 0.88)	0.65 (0.42, 1.01)
35–45	19 (3.8)	483 (96.2)	0.37 (0.21, 0.65)	0.45 (0.25, 0.83)
CD4 count (cells/mm^3^)				
< 200	13 (2.9)	433 (97.1)	Ref.	Ref.
200–350	23 (4.3)	516 (95.7)	1.48 (0.74, 2.97)	1.32 (0.65, 2.67)
> 350	62 (6.1)	958 (93.9)	2.16 (1.17, 3.96)	1.89 (1.01, 3.53)
Missing	33 (19.4)	137 (80.6)	8.02 (4.11, 15.70)	7.62 (3.83, 15.20)
WHO clinical stage				
I	87 (6.3)	1302 (93.7)	Ref.	Ref.
II	26 (5.7)	433 (94.3)	0.90 (0.57, 1.41)	1.00 (0.63, 1.60)
III	9 (3.6)	243 (96.4)	0.55 (0.28, 1.12)	0.85 (0.41, 1.76)
IV	3 (5.0)	57 (95.0)	0.79 (0.24, 2.57)	0.81 (0.24, 2.77)
Missing	6 (40.0)	9 (60.0)	9.98 (3.47, 28.70)	7.84 (2.52, 24.40)
Year of enrollment				
2007–2009	64 (6.5)	918 (93.5)	Ref.	Ref.
2010–2013	67 (5.6)	1126 (94.4)	0.85 (0.60, 1.22)	0.93 (0.64, 1.34)

OR = odds ratio.

CI = confidence interval.

WHO = World Health Organization.

Ref = referent category.

* Patients were classified as having no follow-up if they did not return to care after the initial enrollment visit and otherwise were classified as followed-up.

### Loss to care

**Fig 2:**
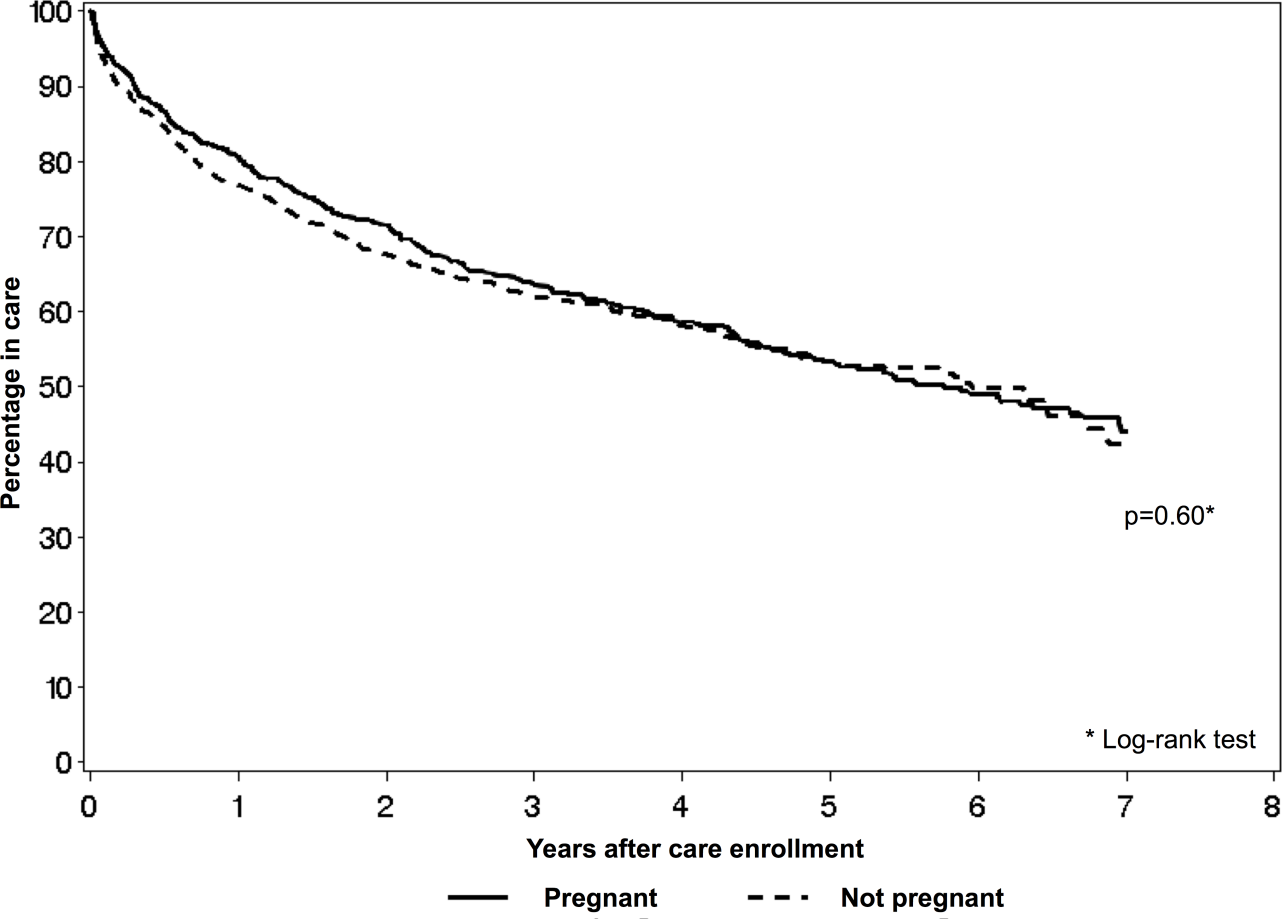
Kaplan-Meier curves for time to loss to care among those who returned for at least one post-enrollment visit, by pregnancy status at enrollment (N = 2044). Includes participants who had ≥ 1 follow-up visit after enrollment. Pregnancy status was recorded at enrollment into care. Participants were classified as lost to care (on their last clinic visit) if > 365 days had passed since their last clinic visit. All participants who did not experience the outcome were censored at the date of last clinic visit or when they were no longer at risk for loss to care (365 days before the database closing date).

We included the 2044 women who returned for at least one post-enrollment visit in the analysis of loss to care (**Fig 2**). Median follow-up time among those patients was 4.5 years (IQR 3.0–5.8), and cumulative proportions of pregnant and non-pregnant patients lost to care at 1, 2, 3, 4 and 5 years after enrollment were as follows: for pregnant patients, 23.1% (95% CI 20.9–25.4%), 32.3% (95% CI 29.9–35.0%), 37.9% (95% CI 35.3–40.7%), 41.8% (95%CI 39.1–44.6%), and 46.5% (95%CI 43.5–49.7%); for non-pregnant patients, 19.2% (95%CI 16.3–22.5%), 28.5% (95% CI 25.1–32.3%), 36.3% (95%CI 32.5–40.4%), 41.1% (95% CI 37.1–45.4%), and 46.7% (95% CI 42.4–51.2%), respectively. In bivariate analysis, loss to care did not differ significantly between pregnant and non-pregnant women (HR 1.04, 95% CI 0.90–1.20; *p* = 0.6 for log-rank test). When adjusted for baseline characteristics and starting ART within three months after enrollment, hazards of loss to care remained similar for the two groups (adjusted HR for pregnant women versus non-pregnant women 1.08, 95% CI 0.93–1.26) (**Table 3**). Sensitivity analyses using a 182-day definition for loss to care demonstrated similar results for the entire follow-up period (aHR 1.09, 95% CI 0.95–1.25) as well as when follow-up time was limited to 24 months after enrollment (aHR 1.19, 95% CI 0.99–1.43).

**Table 3:** Associations of demographic and clinical characteristics at enrollment and loss to care among HIV-infected women with at least one visit after enrollment in care (N = 2044).

Characteristic	Lost to care (n = 860)	Not lost to care (n = 1184)	Crude HR* (95% CI)	* Adjusted HR (95% CI)
Pregnant				
No	288 (44.1)	365 (55.9)	Ref.	Ref.
Yes	572 (41.1)	819 (58.9)	1.04 (0.90, 1.20)	1.08 (0.93, 1.26)
Age (years)				
15–24	176 (52.4)	160 (47.6)	Ref.	Ref.
25–34	521 (42.5)	704 (57.5)	0.72 (0.60, 0.85)	0.76 (0.64, 0.90)
35–45	163 (33.8)	320 (66.3)	0.52 (0.42, 0.65)	0.57 (0.46, 0.71)
CD4 count (cells/mm^3^)				
< 200	145 (33.5)	288 (66.5)	Ref.	Ref.
200–350	194 (37.6)	322 (62.4)	1.10 (0.89, 1.36)	1.08 (0.87, 1.34)
> 350	446 (46.6)	512 (53.4)	1.39 (1.15, 1.67)	1.31 (1.08, 1.58)
Missing	75 (54.8)	62 (45.3)	1.99 (1.50, 2.63)	2.05 (1.54, 2.72)
WHO clinical stage				
I	574 (44.1)	728 (55.9)	Ref.	Ref.
II	176 (40.7)	257 (59.4)	0.89 (0.75, 1.05)	0.99 (0.83, 1.17)
III	86 (35.4)	157 (64.6)	0.77 (0.61, 0.97)	0.88 (0.70, 1.12)
IV	18 (31.6)	39 (68.4)	0.73 (0.45, 1.16)	0.76 (0.47, 1.22)
Missing	6 (66.7)	3 (33.3)	1.78 (0.80, 3.98)	1.32 (0.58, 2.99)
Year of enrollment				
2007–2009	376 (41.0)	542 (59.0)	Ref.	Ref.
2010–2013	484 (43.0)	642 (57.0)	1.52 (1.32, 1.75)	1.56 (1.35, 1.80)
ART initiated within 3 months				
No	607 (48.8)	637 (51.2)	Ref.	Ref.
Yes	253 (31.6)	547 (68.4)	0.60 (0.52, 0.69)	0.54 (0.44, 0.65)

HR = hazard ratio.

CI = confidence interval.

WHO = World Health Organization.

ART = antiretroviral therapy.

Ref = referent category.

* HRs calculated from proportional hazards models of time to last clinic visit.

Includes participants who had ≥ 1 follow-up visit after enrollment. Pregnancy status was recorded at enrollment into care. Participants were classified as lost to care (on their last clinic visit) if > 365 days had passed since their last clinic visit. All participants who did not experience the outcome were censored at the date of last clinic visit or when they were no longer at risk for loss to care (365 days before the database closing date).

## Discussion

This analysis of loss to HIV care of Congolese women found that those pregnant at care enrollment were more likely to never return for follow-up. However, among those who attended at least one follow-up visit, there were no statistically significant differences in loss to care by enrollment pregnancy status. This suggests that further efforts to retain women diagnosed with HIV in pregnancy should focus at the earliest point in care delivery, and that once pregnant women are established in care they can be retained in care as well as non-pregnant women.

About 6% of women who enrolled into care never returned for a follow-up visit. This was about twice as likely to occur among pregnant women versus non-pregnant women. Our results are similar to other studies undertaken both prior to and after implementation of Option B+, which found that pregnant women are two to five times more likely to never return after enrollment into care [11–13, 27]. Many women diagnosed with HIV during routine prenatal screening may be overwhelmed by needing to simultaneously process their diagnosis and deal with competing demands of pregnancy [28–30]. Notably, the proportion of women who never returned for a follow-up visit in our study was substantially lower than these other cohorts [11–13, 27], where 20–33% of patients did not return after the enrollment visit. This finding may be due to the fact that most women in our study were referred to the HIV care and treatment sites from affiliated clinics. It is possible that a substantial proportion of HIV infected women referred to those clinics were lost to care prior to the enrollment visit by never acting on the referral.

Among women who attended at least one follow-up visit, we found no difference in the hazards of loss to care between pregnant and non-pregnant women, before and after adjusting for baseline differences between these groups. This result differs somewhat from those reported by Haas, et al [12]. In that study, among pregnant and non-pregnant women attending at least one follow-up visit after enrollment, pregnant women had lower odds of retention at years 1 and 2 after enrollment versus non-pregnant women, a difference that was no longer significant at year 3 of follow-up. The study by Haas, et al compared pregnant women on Option B+ (immediate ART initiation) to non-pregnant women initiating ART because they met immunological criteria. It is possible that the stronger negative effect of pregnancy on retention they reported resulted from differences between pregnant and non-pregnant women with respect to time in care prior to ART initiation: pregnant women were eligible for immediate ART initiation, whereas women initiating ART because of meeting immunological criteria typically needed to be in care at least long enough to be evaluated for ART eligibility. Other studies that did not distinguish between patients with no follow-up and those who attended at least one follow-up visit have also reported higher loss to follow-up among pregnant women initiating ART compared to non-pregnant women [31–33]. Similar to the Haas et al. study, in these studies, follow-up for non-pregnant women started at ART initiation, inducing a left-censoring bias (any loss to follow-up that occurred between enrollment and ART initiation was not included in the analyses). Our analysis attempts to control for this bias by counting follow-up time in both groups from enrollment and is more relevant to the current context of test-and-treat in which all HIV-infected patients are expected to start ART immediately.

While few studies have described long-term loss to care of pregnant women in HIV care, Haas et al. reported that over 30% of pregnant women were lost at three years after enrollment [12]. Similarly, in our study, we observed that loss to care was high for both pregnant and non-pregnant women, with nearly half the cohort lost to follow-up at five years after enrollment. We further observed that older age, more advanced HIV, and starting ART within three months of enrollment were associated with a lower hazard of loss to follow-up, consistent with findings from other studies [34–36]. Feeling healthy, stigma and anxiety about lifelong treatment are established barriers to retention in HIV care, and may explain these results [37, 38]. Among women enrolling in HIV care while pregnant, specific barriers to retention include prioritizing children’s health over their own, non-disclosure to partners, HIV status denial, and economic concerns [39–41]. While few other studies have examined long-term retention in care of pregnant women, attrition of 30–50% of patients initiating care has been observed in cohorts of general populations [42, 43], adolescents [44], and special populations in sub-Saharan Africa [45]. Although our results reflect the challenges of maintaining patients in long-term HIV care, our finding that patients who initiated ART within 3 months of care enrollment were nearly twice as likely to remain in care as those who did not suggests that early ART initiation under universal test and treat may promote greater retention in care than observed to date in sub-Saharan Africa.

This study has several limitations. The cohort consisted of patients from only two clinics in a country with a relatively low prevalence and burden of HIV compared to other sub-Saharan African countries, which may limit the generalizability of our findings. Additionally, we only included women who enrolled in care in these clinics, but were unable to account for women who were referred to the clinics and never enrolled. While some patients were known to have died or transferred to other facilities, it is possible that additional patients unknowingly experienced these outcomes and were misclassified as lost to care. Because we analyzed previously collected data, we were unable to examine additional factors (e.g., income, distance from clinic) that may have influenced outcomes.

## Conclusions

In this large cohort of women enrolled in HIV care in two clinics in Kinshasa, DRC, we found similar rates of loss to care among pregnant and non-pregnant women. These results suggest that pregnancy itself may not be the main driver of poor retention in HIV care but that confounding factors may also be responsible for the high attrition noted during the early implementation of universal treatment of pregnant women living with HIV under Option B+. As universal test and treat is implemented across sub-Saharan Africa, special attention should be paid to the problem of drop-out immediately following enrollment.
